# Non-Surgical Periodontal Therapy Could Improve the Periodontal Inflammatory Status in Patients with Periodontitis and Chronic Hepatitis C

**DOI:** 10.3390/jcm10225275

**Published:** 2021-11-13

**Authors:** Dorin Nicolae Gheorghe, Dora Maria Popescu, Alex Salan, Mihail Virgil Boldeanu, Claudiu Marinel Ionele, Allma Pitru, Adina Turcu-Stiolica, Adrian Camen, Cristina Florescu, Ion Rogoveanu, Petra Surlin

**Affiliations:** 1 Department of Periodontology, Faculty of Dental Medicine, University of Medicine and Pharmacy of Craiova, 200349 Craiova, Romania; dorinngheorghe@gmail.com (D.N.G.); surlinpetra@gmail.com (P.S.); 2 Department of Oral Surgery, Faculty of Dental Medicine, University of Medicine and Pharmacy of Craiova, 200349 Craiova, Romania; alexsalan87@gmail.com (A.S.); adycamen@icloud.com (A.C.); 3 Department of Immunology, Faculty of Medicine, University of Medicine and Pharmacy of Craiova, 200349 Craiova, Romania; 4 Department of Gastroenterology, Faculty of Medicine, University of Medicine and Pharmacy of Craiova, 200349 Craiova, Romania; ioneleclaudiu@gmail.com (C.M.I.); rogoveanu65@gmail.com (I.R.); 5 Department of Oral Pathology, Faculty of Dental Medicine, University of Medicine and Pharmacy of Craiova, 200349 Craiova, Romania; allmapitru75@yahoo.com; 6 Department of Pharmacoeconomics and Statistical Analysis, Faculty of Pharmacy, University of Medicine and Pharmacy of Craiova, 200349 Craiova, Romania; adina.turcu@gmail.com; 7 Department of Internal Medicine and Cardiology, Faculty of Medicine, University of Medicine and Pharmacy of Craiova, 200349 Craiova, Romania; tohaneanu67@yahoo.com

**Keywords:** periodontitis, chronic hepatitis C, non-surgical periodontal therapy, enzyme-linked immunosorbent assay, gingival fluid, pentraxin-3, c-reactive protein

## Abstract

Non-surgical periodontal therapy (NSPT) is the first essential step for the management of any periodontitis patient. This study aims to evaluate the impact of NSPT on pro-inflammatory mediators’ regulation and on clinical parameters in periodontitis patients who suffer from chronic hepatitis C. At baseline, selected patients were clinically evaluated for their periodontal status. A subsequent quantitative assessment of C-reactive protein and pentraxin-3 in samples of gingival fluid was performed by Enzyme-Linked Immunosorbent Assay (ELISA). Afterwards, NSPT was performed. Three months after NSPT, the clinical and ELISA assessments were repeated. The results show an improvement of the clinical parameters in periodontitis patients at the three-month recall. In chronic hepatitis C patients with periodontitis, the gingival fluid levels of pro-inflammatory markers reduced significantly. The targeted markers also expressed significant correlations with the clinical parameters used for the assessment of periodontitis’ severity. The results suggest that, while chronic hepatitis C patients exhibited a more negative periodontal status at baseline as compared to non-hepatitis ones, NSPT is effective in decreasing the local periodontal inflammatory reaction and in proving the periodontal status of this type of patients. Given the limitation of the study, periodontal screening and NSPT should be included in the integrated therapeutical approach of chronic hepatitis C patients, for its impact on the local inflammatory response.

## 1. Introduction

Periodontitis is an inflammatory disease caused by the subgingival accumulation and growth of bacterial biofilm [1]. The oral cavity hosts over 500 bacterial species, but these are found in a state of equilibrium, called eubiosis, in which commensal bacteria do not allow pathogenic bacteria to cause infection [2]. Periodontitis is triggered when the accumulated subgingival bacterial biofilm is left undisrupted for extended time periods, which allows its colonization with highly pathogenic bacteria [3]. Consequently, these bacteria and their toxins enter the gingival tissues, triggering the inflammatory reaction which characterizes periodontitis [4].

Unfortunately, periodontitis’ consequences extend further than the local disruption of normal dental functions. The periodontium exhibits extended vascular, nervous and lymphatic connections with the rest of the body [5]. Thus, the periodontal status can also be influenced by any pathologic alteration of the general homeostasis [6]. Conversely, periodontitis can also influence the systemic health status of a patient and the clinical manifestation of certain diseases [7]. This bi-directional relationship of periodontitis with systemic health and disease has been studied over the last decade, leading to the development of the “periodontal medicine” concept [8]. This concept comprises and explains the mutual influencing connections that exist between periodontitis and systemic conditions, including diabetes mellitus, cardiovascular diseases and others [9]. The concept is also taken into account by the new 2018 classification of periodontal diseases, stating that, for example, a poorly controlled diabetes mellitus can significantly modify the staging and grading of periodontitis, in terms of severity and rate of progression [10].

Chronic hepatitis C (CHC) is a consequence of the patient’s infection with the Hepatitis C Virus (HCV) and is a major threat for the individual’s life, with an estimated 700,000 world-wide annual deaths [11]. These are usually caused by CHC’s complications, including hepatic cirrhosis and hepatocellular carcinoma [12]. After the infectious contact with the virus, the disease is clinically manifested in only 15% of the cases [13]. In the majority of situations, the initial inflammatory reaction caused by the presence of the virus is “chronicized”, leading to CHC, the virus still being detectable six months after the infectious contact [14]. If left untreated, CHC is a potentially life-threating condition, leading to dangerous complications such as hepatic cirrhosis (in 10–30% CHC patients) [15] and hepatocellular carcinoma (5% of CHC patients) [16].

The gingival crevicular fluid (GCF) is a relevant and easy-to-sample biologic product for the detection of pro-inflammatory mediators, that have been shown to closely followthe severity and intensity of the local periodontal inflammatory reaction, in terms of their quantitative analysis [17]. The study of pro-inflammatory cytokines (interleukin-1alpha and -1beta) in GCF samples has revealed that their levels were significantly up-regulated in CHC periodontitis patients, compared to non-CHC ones [18].

Pro-inflammatory mediators are molecules that trigger, drive, and fuel the inflammatory reaction and include a vast array of elements, such as interleukins, tumor necrosis factors, prostaglandins, thrombin etc. [19]. Pentraxins (PTX) are a family of proteins that are involved in inflammatory mechanisms, acting as pattern-recognition receptors (PRRs) [19]. Also known as acute-phase proteins (APP), PTX play an important role in the onset of inflammation, being mainly involved in protection against pathogenic bacteria, through complement activation [20]. C-reactive protein (CRP) is a type of short pentraxin, while pentraxin-3 (PTX3) is a member of the long pentraxin branch. The two proteins are both-upregulated in severe infections and inflammation, hence their relevance for bacterial generated disease (such as periodontitis) or viral ones (such as CHC) [21].

Given the scientific background and previously published results, the authors aim to develop the hypothesis of existing pathologic connections between periodontitis and CHC, by assessing the influence that non-surgical/initial periodontal treatment (NSPT) might have on the local periodontal inflammatory status of CHC periodontitis patients. This assessment implies the quantitative analysis of the GCF levels of targeted pro-inflammatory mediators (CRP and PTX3), before and after non-surgical periodontal treatment for the local periodontal inflammatory status. Significant differences of the measured parameters could suggest that non-surgical periodontal therapy has a relevant impact on the local homeostasis of CHC periodontitis patients.

## 2. Materials and Methods

### 2.1. Study Design

The authors requested and obtained permission for the study’s implementation from the Ethical Research Committees of the University of Medicine and Pharmacy of Craiova, Romania (no. 128/2021). The study strictly followed the European Union’s General Data Protection Regulation on patient data protection and discretion (GDPR) and the 1975–2003 Declaration of Helsinki. The design of the study included three main stages: (i) Baseline-Initial periodontal status assessment, GCF sampling (for CRP and PTX3 quantitative analysis); (ii) non-surgical periodontal treatment NSPT (as indicated by the periodontal evaluation); (iii) Recall-reassessment of periodontal status and resampling of GCF samples. The resulted data was afterwards used for statistical analysis. The design of the study was constructed based on similar studies, performed in periodontitis patients with systemic illnesses, such as type-2 diabetes mellitus or rheumatoid arthritis [22,23].

### 2.2. Patient Selection

The study aimed at selecting three categories of participants, addressing the Periodontology and Gastroenterology departments of the University of Medicine and Pharmacy of Craiova: (i) CHC patients with periodontitis; (ii) periodontitis patients (non-CHC); (iii) controls (non-CHC, non-periodontitis).

The selection criteria for CHC patients consisted of: (i) asymptomatic forms of CHC; (ii) no detectable HCV viremia in the last six months. The selection criteria used for the inclusion of periodontitis patients followed the 2018 Classification of Periodontal Disease guidelines and consisted of: (i) periodontal pockets ≥ 4 mms in more than 30% of teeth; (ii) interdental recession. This resulted in Stage II and III periodontitis diagnosis status (Moderate to Severe Periodontitis). Control patients were included in the study after showing no sings, symptoms, or history of either systemic or periodontal diseases. Other general exclusion criteria were: (i) active smoking status; (ii) anti-inflammatory or antibiotic medication in the last 30 days prior to initial sampling of GCF; (iii) previous antiviral anti-HCV therapy; (iv) pregnancy.

Written informed consent was obtained from all selected patients. In total, a number of 44 participants was comprised, divided into three study groups: (i) HCV + P group: 15 patients (aged from 39 to 74 years) with confirmed CHC and periodontitis (P); (ii) P group: 17 patients (aged from 36 to 72 years) with confirmed periodontitis; (iii) H group: 12 healthy controls (aged from 41 to 68 years) with no periodontitis, no chronic hepatitis C.

### 2.3. Periodontal Assessment

The periodontal assessment of all participating patients was performed with the use of a manual periodontal probe (University of North Carolina probes, Medesy, Maniago, Italy) by the same, calibrated, evaluator. All existing teeth were probed into six points (mesial, central, and distal for the buccal and oral sides of the teeth). The data generated by the periodontal probing (sulcus/periodontal pocket depth, position of the marginal gingival in relation to the cementoenamel junction, presence of plaque deposits, bleeding points, and mobility) were inserted into the digital periodontal chart issued by the University of Bern (www.periodontalchart-online.com; last accessed 3 September 2021). For each patient, the digital chart automatically computed the values of the following periodontal parameters: (i) mean probing depth (mms); (ii) mean attachment level (mms); (iii) plaque index (%); and (iv) bleeding on probing index (%).

### 2.4. Gingival Crevicular Fluid Sampling

Gingival crevicular fluid samples were collected from each participant, using absorbent paper strips (PerioPaper, Oraflow Inc., Smithtown, NY, USA). For the sampling procedure, the two teeth with the deepest periodontal pocket/sulcus were chosen. Preventive measures for sample contamination were taken, such as the use of cotton rolls and air suction (for no contact with saliva). Absorbent paper strips were inserted simultaneously at the two targeted teeth for sampling. The strips were kept in place for 30 s. Consequent to their removal from the periodontal pocket, they were visually inspected for lack of bloodstains, and were subjected to GCF quantity standardization using the Periotron 8000 device (Oraflow Inc., Smithtown, NY, USA). The resulted two paper strips were afterwards placed separately into polyethylene microtubes with saline buffer solution (PBS). The microtubes were preserved at −80 degrees Celsius, until sampling completion.

### 2.5. Immunological Assessment

After GCF samples had been collected, the quantitative assessment of the targeted pro-inflammatory mediators (CRP and PTX3) was performed by the Immunology Laboratory of the University of Medicine and Pharmacy of Craiova, through enzyme-linked immunosorbent assay (ELISA). Commercial kits were used for each mediator, Quantikine DPTX30 Human PTX-3 ELISA Kit, R&D Systems (Minneapolis, Minesota, USA; range 0.3–20 ng/mL) and Invitrogen CRP Human ELISA Kit, ThermoFisher Scientific (Waltham, Massachusetts, USA; range 18.75–1200 pg/mL) according to the manufacturer’s indications and prescribed method. A standard optical analyzer at 450 nm wavelength was used during the process.

### 2.6. Non-Surgical Periodontal Therapy-NSPT

As generated by the periodontal screening and assessment, all participating patients, apart from those of control group, were included in the Stage II and III periodontitis category. This implied that they accepted and followed standard non-surgical periodontal therapy, which included the therapeutic sequence: (i) full mouth ultrasonic scaling and gingival debridement (EMS, Nyon, Switzerland); (ii) full mouth air-flow polishing (EMS, Nyon, Switzerland); (iii) subgingival irrigations with Metronidazole and Chlorhexidine solutions (Detax, Ettlingen, Germany); (iv) manual scaling of periodontal pockets using Gracey curettes (Medesy, Maniago, Italy). Complementary to the in-office treatment, patients were also instructed to implement the following at-home procedure: (i) morning and evening teeth brushing; (ii) use of interdental brushes; (iii) oral rinsing with 0.12% Chlorhexidine mouthwash (Curasept, Curaprox, Bologna, Italy).

### 2.7. Recall–Periodontal Reassessment and Resampling of Biological Products

After 90 days from NSPT, the patients were recalled for revaluation of their periodontal status and sampling of biological products. The periodontal probing was reperformed on all teeth, as well as the digital periodontal chart. Consequently, GCF was sampled from the same two teeth as the first time. The post-NSPT GCF samples were transferred to the Immunology Laboratory, for the CRP/PTX3 ELISA analysis.

### 2.8. Statistical Analysis

Data were analyzed using GraphPad Prism 9.2.0 (GraphPad Software, San Diego, CA, USA). Continuous variables were compared using Mann-Whitney test due to the small number of included patients. The correlation heatmap was created to assess the Spearman correlations between baseline and after NSPT characteristics. Violin plots were created (box plots also showing the probability density of the data at different values) to visually compare measured parameters between baseline and after NSPT. The power analysis for our study was performed using G*Power 3.1.9.7, at a 95% confidence level and power factor of 80% for each of the groups. A two-sided *p*-value smaller than 0.05 was considered to be statistically significant. The power test was performed assuming an alpha level of 0.05, the patients from HCV+P and P groups yielded the power between 66% and 85% for the different analyzes.

## 3. Results

### 3.1. Baseline Status

The baseline periodontal status of the groups shows that the mean probing depth was significantly more elevated in HCV+P patients, as compared to P ones (*p* = 0.0247). In HCV+P patients, the bleeding on probing was also significantly higher than in P ones (*p* < 0.0001), despite similar levels of plaque index for the two groups (*p* = 0.9479). The GCF CRP value was higher for the HCV+P group than the P group, but not statistically significant (*p* = 0.1243). However, these values were significantly elevated in the two groups of patients with periodontitis, as compared to healthy controls (*p* < 0.0001). (Table 1). The GCF PTX3 levels were highest in the HCV+P group, the difference being statistically significant to the P (*p* = 0.0307) and H (*p* < 0.001) groups. The GCF PTX3 levels were also significantly more elevated in the P group, as compared to healthy controls. (Table 1).

**Figure 1:**
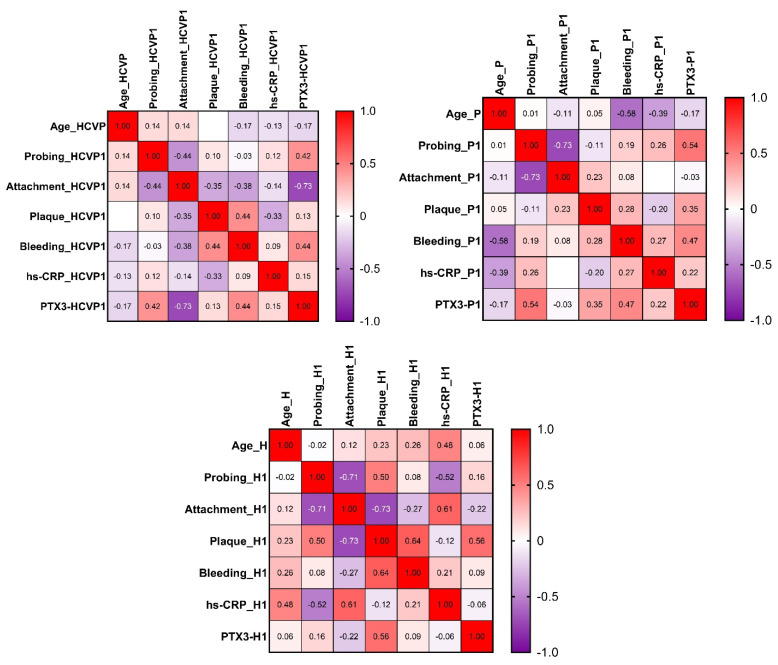
Heatmap of the correlation matrix for every group of patients before the treatment: HCVP1 = HCV+P group at baseline; P1 = P group at baseline; H1 = H group at baseline; hs-CRP = high-sensitivity C-reactive protein; PTX3 = pentraxin-3.

The strength of the correlation between the characteristics of the patients from the three groups before the treatment is represented by the color of the square at the intersection of those variables from the heatmap, as shown in Figure 1. Colors range from bright red (strong positive correlation; r = 1.0) to bright mauve (strong negative correlation; r = −1.0). For the HCV+P group, Spearman’s correlation analysis revealed significantly negative correlation between PTX3 and attachment levels (r = −0.732, *p*-value = 0.003). For the P group, significantly negative correlations were found between age and bleeding index (r = −0.578, *p*-value = 0.017), and attachment levels and probing depths (r = −0.733, *p*-value = 0.001). A significantly positive correlation was found between probing depths and PTX3 (r = 0.537, *p*-value = 0.028).

### 3.2. Recall—After NSPT Status

The revaluation performed 3 months after NSPT saw an improvement of periodontal clinical parameters in both HCV+P and P groups. The mean pocket depth parameter decreased in both groups, the difference between them not being significant anymore (*p* = 0.0536). Between the two groups of periodontitis patients, the difference in attachment loss was still statistically significant (*p* = 0.0285). Although the bleeding on probing index remained higher for the HCV+P group than for the P one, the difference was not statistically significant (*p* = 0.233), similar to the plaque index situation (0.9331). All evaluated indexes remained significantly modified as compared to healthy controls. (Table 2)

The GCF CRP levels reduced 3 months after NSPT in the HCV+P group, and increasing slightly for the P group. In both groups, the GCF CRP levels remained significantly elevated, as compared to healthy controls (*p* < 0.001). (Table 2). The GCF value of the PTX3 marker reduced in both the HCV+P group and the P group, the difference between the two groups remaining statistically significant (*p* = 0.0392), as well as compared to the value of the H group (*p* < 0.001). (Table 2).

**Figure 2:**
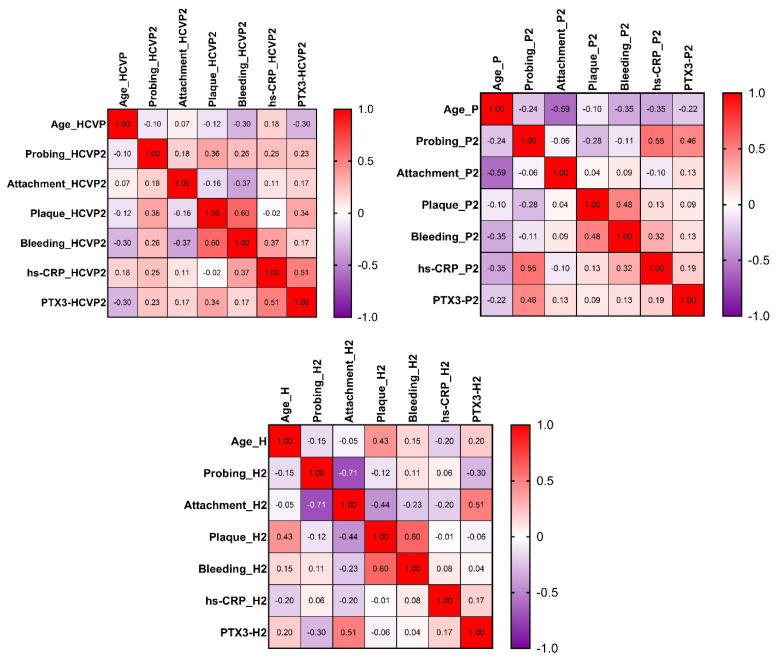
Heatmap of the correlation matrix for every group of patients after the treatment: HCVP2 = HCV+P group at recall; P2 = P group at recall; H2 = H group at recall.

For the HCV+P group, Spearman’s correlation analysis after treatment revealed significant positive correlation between bleeding and plaque indexes (r = 0.600, *p*-value = 0.020). For the P group, a significant negative correlation was found between age and attachment levels (r = −0.593, *p*-value = 0.014), and a significant positive correlation between probing and hs-CRP (r = 0.548, *p*-value = 0.024), as shown in Figure 2.

### 3.3. Baseline vs. Recall, after-NSPT Status Differences

**Figure 3:**
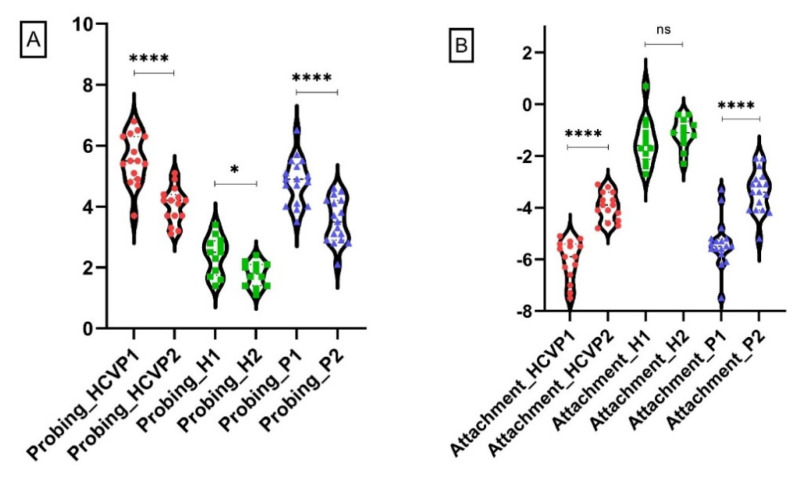

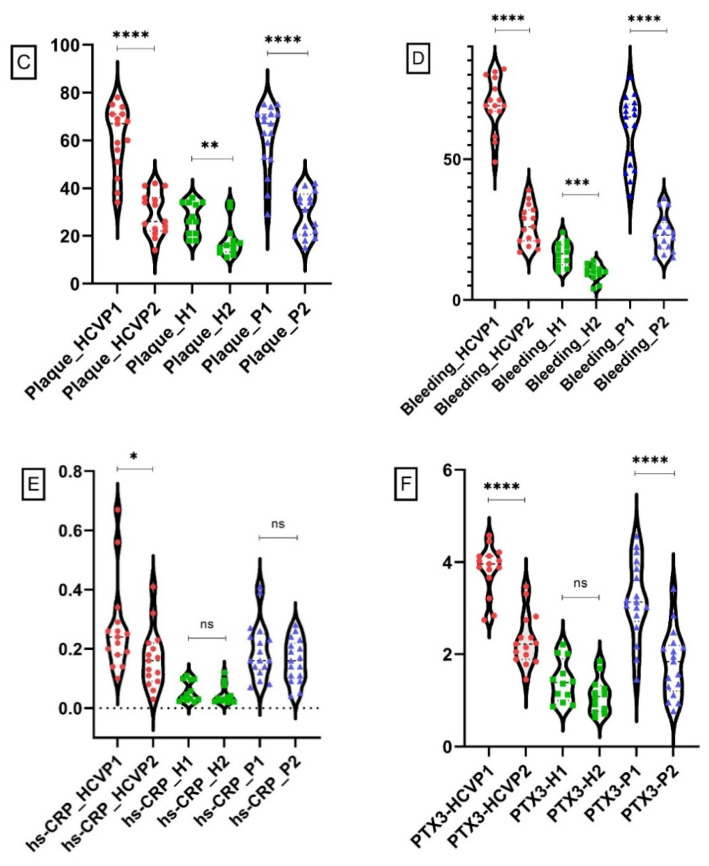
Levels of clinical and immunologic parameters baseline vs. recall. HCVP1 = HCV+P group baseline; HCVP2 = HCV group recall; P1 = P group baseline; P2 = P group recall; H1 = H group baseline; H2 = H group recall. (**A**) Mean probing depth. (**B**) Mean attachment level. (**C**) Plaque. (**D**) Bleeding on probing. (**E**) hs-CRP. (**F**) PTX3. Data were analyzed for statistical significance using Mann-Whitney tests between groups. *, *p* < 0.05; **, *p* < 0.01; ***, *p* < 0.001; ****, *p* < 0.0001; ns = not significantly different.

The comparison of the baseline and after-NSPT results (Figure 3) shows a significant improvement of all used periodontal parameters for the HCV+P group (*p* < 0.001). In addition, the targeted mediators, CRP (*p* = 0.0191) and PTX3 (*p* < 0.0001), also expressed significantly reduced GCF levels (Table 3). For the P group, NSPT produced a significant improvement of clinical periodontal parameters and a significant decrease of the GCF PTX3 level (*p* < 0.0001). The slight increase of GCF CRP levels was not statistically significant for this group, 3-months after NSPT (*p* = 0.3978). (Table 3). For the control group, the 3-month reevaluation showed a significant change in plaque (*p* = 0.0019) and gingival bleeding indexes (*p* = 0.0003) (Table 3; Figure 3).

As shown in Table 4, most assessed parameters (clinical and immunological) showed a similar evolution in the groups of patients with periodontitis (HCV+P and P), from baseline to recall, apart from the GCF PTX3 parameter, which expressed a more important decrease in P group (−43.75%), than in the HCV+P one (−39.47%).

## 4. Discussion

Periodontitis and CHC are characterized by chronic inflammatory reactions, which can mainly lead to the accumulation of their clinical consequences in adult patients. In our study, there was no statistical difference between the ages of the three groups, therefore with no significant influence on its results. At the baseline evaluation, the PTX3 pro-inflammatory mediator expressed the highest levels in samples of the HCV+P group, the difference to the other groups, being significant. The mediator also correlated with clinical parameters used for the assessment of periodontitis’ severity, such as the mean probing depth, attachment level, and bleeding on probing index. These results are similar to those of other authors [20]. In a 2011 paper, Pradeep et al. assessed the expression of the PTX3 mediator in GCF samples of patients with periodontal disease, showing that its highest levels were in samples originating from periodontitis cases [20]. The clinical periodontal parameters used by the authors also correlated with the GCF PTX3 levels, the authors assuming that as neutrophil cells arrive early at the spot of bacterial aggression, they release significant volumes of this mediator. It is known that in neutrophil cells, PTX3 is pre-stored, in order to be available for rapid release and activity. In the same paper, Pradeep et al. conclude that the GCF PTX3 levels elevate proportionately with the severity of periodontitis [20].

The recall evaluation showed that GCF PTX3 levels decreased after NSPT in both periodontitis groups, although the decrease for the P group (−43,75%) was superior to that of the HCV+P group (−39,47%). This difference in reduction, despite similar improvements of clinical parameters could be explained by the co-existing hepatic inflammation in HCV+P patients, as compared to P ones, that might still generate elevated levels of pro-inflammatory mediators in these patients. Both periodontitis groups exhibited a significant improvement of the assessed periodontal parameters, three months after NSPT. Similar results are reported by Mohan et al., who evaluated, in a 2018 paper, the effect of NSPT on PTX3 GCF levels in patients with periodontitis [23]. The authors found that, at baseline, the GCF PTX3 levels were highest in patients with periodontitis, as compared to controls, and that these levels were significantly reduced as soon as two weeks after performing scaling and root planning on these patients. However, the authors observed a greater reduction of GCF PTX3 levels in smoking patients, suggesting a significant impact of smoking on this parameter. The same authors also found that, in smoking patients, GCF PTX3 levels also remained elevated even after NSPT. Nevertheless, in our study, no smoking patients were included.

The GCF PTX3 levels also correlated with plaque levels in periodontitis patients, similar to a 2014 paper by Gümüş et al. that evaluated the PTX3 levels in saliva samples of aggressive or chronic periodontitis, showing that the mediator expressed significantly elevated levels in saliva samples of aggressive periodontitis patients [24]. These levels also correlated with certain clinical parameters, including the Plaque and Bleeding on Probing Index. Thus, the authors concluded that PTX3 levels could be correlated with the level of periodontal tissue inflammation. Similar results were reached by a 2012 article of Fujita et al., stating a strong correlation between GCF PTX3 level and the periodontal status, and suggesting the use of it as a useful diagnostic marker for periodontal disease [25]. This statement is supported by the high concentration of PTX3 in GCF samples originating from periodontal lesions, as compared to healthy sites and its correlation with clinical parameters such as gingival index, periodontal pocket depth, and the bleeding on probing index.

Our results indicate that PTX3 has good correlations with the most frequently used clinical parameters for periodontal evaluation: mean probing depth, mean attachment level, bleeding on probing and plaque index. In periodontitis patients, the GCF levels are significantly increased than in controls, but they show a relevant reduction after such patients undergo NSPT. Thus, the quantitative assessment of PTX3 by ELISA methods could be used as an adjuvant-testing tool for periodontitis. In addition to this, a 2016 paper by Çalapkorur et al., proposed GCF PTX3 levels as a diagnostic and prognostic marker for periodontitis [26]. This was based on the findings that PTX3 is locally secreted at the sites of inflamed gingival tissues and that its levels correlate with the disease severity. This hypothesis is also supported by a 2017 study by Temelli et al., who showed that the periodontal inflamed surface area (PISA) positively correlated with serum PTX3 levels [27]. However, their research found no significant difference for GCF PTX3 levels between patients with periodontitis and with/without a systemic disease, in this case peripheral arterial disease. This may suggest that GCF PTX3 levels are independent of systemic illness concerning periodontitis patients. Despite this hypothesis, according to Nerkiz et al., serum PTX3 levels were suggested as “novel diagnosis predictors” for coronary artery disease patients [28].

To our knowledge, this is the first study to evaluate the behavior of post-NSPT GCF pro-inflammatory mediators, by the ELISA method, in patients with periodontitis and chronic hepatitis C. Tasdemir et al. evaluated the effect of NSPT on serum PTX3 levels in patients with periodontitis and other systemic diseases, such as diabetes mellitus and chronic kidney disease [22]. The authors’ findings show that NSPT improved all periodontal clinical parameters in all participating patients, 3 months after NSPT. Serum PTX3 levels were also significantly elevated at baseline in periodontitis + diabetic neuropathy patients, as compared to the other groups. In the same group of patients, the serum PTX3 levels significantly reduced 3 months after performing the NSPT. Similar results were reported by Mathew et al., in a 2015 paper on the evaluation of PTX3 levels in chronic periodontitis patients, before and one-month after treatment [29]. The same authors [30] published a paper, in which they evaluated GCF PTX3 levels, before and after NSPT in generalized periodontitis patients, alongside the use of adjuvant ozone therapy. The authors report an improvement of periodontal clinical parameters at a 3-month period to NSPT, compared to baseline, correlated with a significant reduction in GCF PTX3 levels.

In our study, the GCF CRP levels showed less correlation power and therefore, less periodontal clinical relevance than the PTX3 mediator. GCF, CRP levels were significantly elevated in samples of periodontitis patients, as compared to healthy controls. However, there was no statistical difference of the marker’s levels in periodontitis patients with and without chronic hepatitis C. After NSPT the GCF CRP levels decreased in HCV+P patients but showed a contradicting slight increase in periodontitis-only patients. A comparable reduction of GCF CRP levels was reported by Mohan et al. [23]. The authors also reported a greater significant reduction of periodontal clinical parameters in patients suffering from type-2 diabetes mellitus than in non-diabetic patients. The authors also found a significant positive correlation between CRP levels and periodontal clinical parameters. However, the authors state that it was not clear if elevated CRP levels were attributed only to periodontal inflammation [23].

The GCF CRP levels correlated with age and the plaque index, suggesting that it may be more relevant to more elderly patients, who tend to have more precarious oral hygiene. The GCF CRP levels also correlated with the bleeding on probing index. These parameters correspond to the profile of the CHC patients, who, in this study showed significantly more bleeding on probing than non-CHC ones. This fact can be hypothesized with the hepatic nature of CRP, which, in contrast to PTX3, is not locally pre-synthesized and may require longer period of time to reach relevant concentrations in the GCF. This idea was also suggested by a study by Singh et al., which found that, while NSPT produced a significant improvement of periodontal clinical parameters, such as plaque index (due to improvement of oral hygiene) and pocket depth/attachment loss (due to reduction of inflammation), the serum CRP levels failed to decrease significantly, after a one- and two-month recall [31]. The authors suggest that this was due to the small number of participants or short period recall [31].

Regarding the association of periodontitis and systemic disease, Bian et al. assessed the influence of NSPT on CRP levels in patients with periodontitis and type-2 diabetes mellitus [32]. Our study showed that 3 months after NSPT, CRP levels decreased significantly, suggesting that CRP is also influenced by liver stress status. A study by Baser et al. found significant positive correlation between CRP levels and the severity of periodontitis in patients with type-2 diabetes mellitus [33]. A review paper by Sun et al. concluded that NSPT significantly reduced CRP serum levels in periodontitis patients with rheumatoid arthritis [34]. Our study reached comparable results, showing that in HCV+P patients, NSPT significantly reduced the GCF CRP levels (*p* = 0.0191) at a 3-month recall. In patients with viral hepatic infection, studies have shown that vitamin D may have a significant suppressor anti-viral effect [35]. However, in periodontitis patients, the levels of salivary vitamin D are decreased, as compared to controls [36], suggesting that, in the case of HCV+P patients, the protective anti-viral role of vitamin D may be downregulated. This hypothesis would suggest a more increased viral activity and a consequent inflammatory reaction in HCV+P patients. 

The limitations of this study stem from the reduced number of participating patients [37], generated by the numerous inclusion and exclusion criteria required for an objective assessment. Given the limited time span of the study, future additional research will be carried on the subject in order to evaluate extensively the effects on NSPT in periodontitis patients with chronic hepatitis C. For the moment, our results indicate that NSPT produces a significant improvement of the clinical and immunological status of HCV+P patients, as shown clinically and by means of ELISA assessment. Future developments of this study would also address patients with dental implants, as mucositis and periimplantitis have recently increased in prevalence and require particular therapeutical means, especially in patients with systemic diseases [38].

NSPT produced a significant improvement of the clinical periodontal status of periodontitis patients with chronic hepatitis C, as reflected by the clinical periodontal examination and ELISA assessment of the GCF PTX3 and CRP levels. Following NSPT, a significant reduction of the GCF PTX3 levels was identified, although of a lesser magnitude than in periodontitis patients with no chronic hepatitis C. This finding suggests a possible involvement of the hepatic pathology in the local inflammatory processes of periodontitis, that requires further, future research, similar to a broader approach on the role of the CRP mediator in a common periodontitis-hepatitis C scenario.

## 5. Conclusions

As highlighted by the ELISA analysis of the PTX3 and CRP markers in gingival fluid samples, it could be said that non-surgical periodontal therapy has a relevant, improving, impact on the local homeostasis of periodontitis + hepatitis C patients.

## Figures and Tables

**Table 1:** Average ± standard deviation and statistical significance of analyzed parameters before treatment.

Parameter	HCV+P (*n* = 15)	P (*n* = 17)	H (*n* = 12)	*p*-Value HCV+P vs. P	*p*-Value HCV+P vs. H	*p*-Value P vs. H
Age (years)	50.8 ± 8.7	51.4 ± 11.3	52.4 ± 9.6	0.8011	0.5552	0.6704
Mean probing depth (mms)	5.5 ± 0.8	4.8 ± 0.8	2.4 ± 0.6	0.0247	<0.0001	<0.0001
Mean attachment level (mms)	−6.0 ± 0.8	−5.4 ± 0.9	−1.4 ± 0.9	0.0797	<0.0001	<0.0001
Plaque (%)	61 ± 13.8	61.3 ± 13.9	27.4 ± 6.7	0.9479	<0.0001	<0.0001
Bleeding on probing (%)	69.5 ± 9.5	60.2 ± 12.5	16.3 ± 4.3	<0.0001	<0.0001	<0.0001
hs-CRP (ng/mL)	0.3 ± 0.2	0.2 ± 0.1	0.1 ± 0.03	0.1243	<0.0001	<0.0001
PTX3 (ng/mL)	3.8 ± 0.5	3.2 ± 0.9	1.4 ± 0.5	0.0307	<0.0001	<0.0001

**Table 2:** Average ± standard deviation and statistical significance of analyzed parameters after treatment.

Parameter	HCV+P (*n* = 15)	P (*n* = 17)	H (*n* = 12)	*p*-Value HCV+P vs. P	*p*-Value HCV+P vs. H	*p*-Value P vs. H
Age (years)	50.8 ± 8.7	51.4 ± 11.3	52.4 ± 9.6	0.8011	0.5552	0.6704
Mean probing depth (mms)	4.0 ± 0.6	3.5 ± 0.7	1.8 ± 0.4	0.0536	<0.0001	<0.0001
Mean attachment level (mms)	−3.9 ± 0.5	−3.3 ± 0.8	−1.1 ± 0.6	0.0285	<0.0001	<0.0001
Plaque (%)	29.1 ± 8.9	29.2 ± 9.1	18.2 ± 7.4	0.9331	0.0010	0.0006
Bleeding on probing (%)	26.6 ± 6.9	23.5 ± 6.7	9.7 ± 2.9	0.2330	<0.0001	<0.0001
hs-CRP (ng/mL)	0.2 ± 0.1	0.3 ± 0.1	0.05 ± 0.03	0.9036	<0.0001	<0.0001
PTX3 (ng/mL)	2.3 ± 0.6	1.8 ± 0.7	1.1 ± 0.4	0.0392	<0.0001	0.0043

**Table 3:** Differences between characteristics before vs. after treatment.

	HCV+P (*n* = 15) *p*-Value before vs. after	P (*n* = 17) *p*-Value before vs. after	H (*n* = 12) *p*-Value before vs. after
Mean probing depth (mms)	<0.0001	<0.0001	0.0149
Mean attachment level (mms)	<0.0001	<0.0001	0.1817
Plaque (%)	<0.0001	<0.0001	0.0019
Bleeding on probing (%)	<0.0001	<0.0001	0.0003
hs-CRP (ng/mL)	0.0191	0.3978	0.3399
PTX3 (ng/mL)	<0.0001	<0.0001	0.0887

**Table 4:** Evolution (%) of analyzed parameters from baseline to recall.

Parameter	HCV+P (*n* = 15)	P (*n* = 17)	H (*n* = 12)
Mean probing depth (mms)	−27.27	−27.08	−25
Mean attachment level (mms)	−35	−38.88	−21.42
Plaque (%)	−52.29	−52.36	−33.57
Bleeding on probing (%)	−61.72	−60.96	−40.49
hs-CRP (ng/mL)	−33.33	33.33	−50
PTX3 (ng/mL)	−39.47	−43.75	−21.42

## Data Availability

The data used to support the findings of this study are available from the corresponding author upon reasonable request.
